# *Bifidobacterium animalis* subsp. *lactis* TG11 ameliorates loperamide-induced constipation in mice by modulating gut microbiota

**DOI:** 10.3389/fmicb.2025.1525887

**Published:** 2025-01-29

**Authors:** Weiwei Ma, Yiyang Zhao, Yuyang Liu, Yanyan Wang, Shuang Yu, Lili Huang

**Affiliations:** College of Pharmacy, Heilongjiang University of Chinese Medicine, Harbin, China

**Keywords:** probiotics, constipation, gut microbiota, gut peptides, SCF/c-kit

## Abstract

**Introduction:**

Constipation is a common gastrointestinal disorder that can affect quality of life. Probiotics have garnered substantial attention for their potential to alleviate constipation. This study investigates the preventive effects of *Bifidobacterium animalis* subsp. *lactis* TG11 on loperamide-induced constipation in mice.

**Methods:**

Mice were randomly assigned to normal control (NC), constipation model (CM), and low, medium, and high-dose TG11 treatment groups (LG, MG, HG). From days 1–14, LG, MG, and HG groups received 10^6^, 10^7^, and 10^8^ CFU/mouse of TG11, respectively, while NC and CM groups received saline. On day 14, all groups except NC were administered loperamide (4 mg/kg) orally to induce constipation. Fecal samples were collected for short-chain fatty acid and gut microbiota analyses. Following a 16-hour fasting period, various parameters were assessed on day 15, including intestinal motility, fecal water content, defecation status, gut peptide levels in blood, and mRNA expression levels of *SCF* and *c-kit* in colonic tissue.

**Results:**

TG11 significantly enhanced intestinal motility and maintained fecal water content. It normalized blood levels of MTL, SP, SS, ET-1, Gas, and VIP in constipated mice, promoted short-chain fatty acid production, and improved microbial metabolism. TG11 markedly upregulated mRNA expression of *SCF* and *c-kit* in colonic tissue. Metagenomic sequencing revealed that TG11 modulated gut microbiota composition, increasing the abundance of beneficial bacteria, particularly *Muribaculum*_sp. and *uncultured_Duncaniella*.

**Discussion:**

*Bifidobacterium animalis* subsp. *lactis* TG11 demonstrates efficacy in ameliorating constipation, potentially through modulation of the gut microbiota composition.

## Introduction

Constipation is a prevalent disorder of the digestive system characterized by infrequent bowel movements, hard stools, and difficulty in stool passage. Chronic constipation can precipitate anorectal conditions and negatively affect mental health, thus significantly deteriorating life quality (Barberio et al., 2021). Common therapeutic strategies encompass dietary modifications, pharmacological interventions, topical lubricants, enemas, and surgical procedures, each associated with potential adverse effects (Sharma et al., 2021).

The gut microbiota consists of trillions of microorganisms, including bacteria, fungi, and viruses, plays integral roles in digestion, metabolism, immunity, gut barrier integrity, and vitamin production. These microorganisms generate short-chain fatty acids (SCFAs) through the fermentation of indigestible polysaccharides and resistant starch, thereby maintaining gut health (Ding et al., 2024). Dysbiosis, marked by a decrease in beneficial bacteria and an increase in pathogenic counterparts, leads to reduced microbial diversity (DeGruttola et al., 2016). Research indicates a diminished prevalence of beneficial bacteria such as *Bifidobacteria* and *Lactobacilli* in patients with constipation, which compromises gut functionality (Nagpal et al., 2017).

Gut peptides, synthesized by enteroendocrine cells, regulate various physiological processes including digestion and gut motility. Notable peptides include somatostatin (SS), motilin (MTL), gastrin (Gas), substance P (SP), and vasoactive intestinal peptide (VIP), which are pivotal in gastrointestinal function, gut mucosal barrier integrity, and immune modulation. In constipation, abnormal peptide levels can be observed in the bloodstream (Gribble and Reimann, 2019; Xie et al., 2022). The SCF/c-kit signaling pathway is vital for the growth and survival of intestinal smooth muscle cells, enhancing gut contractility and motility. Conversely, its inhibition or dysfunction can diminish these cells or impair their function, leading to weakened peristalsis and constipation onset (Kim et al., 2023; Liu et al., 2021).

Probiotic therapy offers a safer, milder alternative for managing constipation. Studies have demonstrated that probiotics influence constipation by altering gut transit time, stool frequency, and consistency, as well as microbiota composition (Tegegne and Kebede, 2022; Westfall et al., 2021). Recent investigations have highlighted the role of specific *Bifidobacteria* strains in ameliorating constipation through various mechanisms, including dysbiosis correction, SCFA concentration increase, and neurotransmitter regulation (Makizaki et al., 2021; Tang et al., 2022; Wang et al., 2021; Zhang et al., 2023a). For instance, strains such as *Bifidobacterium* G9-1 (BBG9-1) and *Bifidobacterium animalis* subsp. MN-Gup (MN-Gup) have shown promising results in both animal models and human trials.

In this study, we evaluate the protective effects of *Bifidobacterium lactis* TG11 against loperamide-induced constipation in mice, exploring the potential mechanisms through which TG11 mitigates constipation symptoms.

## Materials and methods

### Materials

*Bifidobacterium lactis* TG11, isolated from the intestines of healthy infants, is now maintained by Jinhua Galaxy Biotechnology Co., Ltd., located in China. The preservation is cataloged under the accession number 20230421003. The bacterial activity has been adjusted to 200 billion CFU/g.

### Animal experiments

**FIGURE 1:**
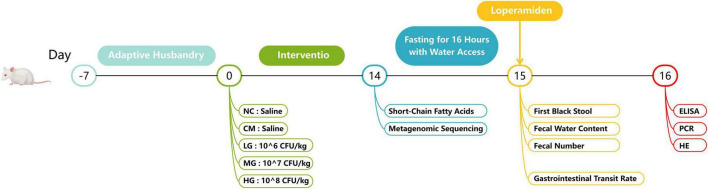
Illustrates the implementation process of the animal experiment conducted in this study.

The research protocol for the animal studies received ethical approval from the Animal Welfare Ethics Review Committee of Heilongjiang University of Chinese Medicine (2024032915, Harbin, Heilongjiang, China). Male ICR mice, with a body mass range of 18–22 grams, were accommodated in a controlled laboratory environment and provided with standard care. The experimental process is detailed in Figure 1. The investigation commenced seven days following the adaptation period. Randomly assigned into five cohorts, the mice included a control cohort (NC), a constipation-induced model cohort (CM), and three treatment cohorts (TG11) with varying dosages: low (LG), medium (MG), and high (HG), each consisting of 20 animals. The trial was bifurcated into two phases: the TG11 treatment phase and the constipation induction phase, guided by the “Functional Assessment and Evaluation Protocols for Health Foods (2023 Edition)” for model design and loperamide dosing.

During the TG11 treatment phase (days 1–14), the NC and CM cohorts received saline solution, while the LG, MG, and HG cohorts were dosed with 10^6, 10^7, and 10^8 CFU/mouse of the bacterial preparation, respectively. On day 14, loperamide was orally administered to the CM cohort and all TG11 treatment cohorts at a rate of 4 mg/kg BW, with the NC cohort continuing to receive saline. The animals in all cohorts were subjected to a 16-h fasting period, maintaining access to water. On day 15, a solution of activated charcoal (5% charcoal and 10% arabic gum) at a volume of 10 ml/kg BW was administered. Ten mice from each group were closely monitored to document the time until the first instance of black stool was observed. Concurrently, an additional cohort of ten mice was assessed for gastrointestinal transit rate (GTR). Throughout the duration of the experiment, the mice were afforded unlimited access to both food and water, and their body weights were consistently recorded at regular intervals.

### Sample collection and analysis

On the 15th day, fecal samples from the groups of mice were collected to measure their fecal water content (FWC). Upon the conclusion of the experiment, blood was drawn and serum was isolated using a refrigerated centrifuge set at 12,000 *g* for 10 min. This serum was then prepared for subsequent biochemical analyses. Samples from both the small intestine and colon were harvested for histological evaluation and real-time PCR analysis. Before oral administration of a solution of activated charcoal, Feces of mice were collected for short-chain fatty acid and metagenomic analysis, respectively.

### Evaluation of time to first black stool and 5-h quantification of stool count and weight

Each mouse was housed in a clean cage and monitored immediately after drug administration. The time to first black stool occurrence was documented. Starting from the first black stool emergence (0 h), mice were continuously observed for 5 h, during which the number and weight of black stools were recorded.

### Determination of fecal water content

The feces of mice in each group were collected for five consecutive hours starting from the first black stool, and wet weight and dry weight are calculated. Immediately after defecation, the feces were weighed while wet. The samples were then placed in an oven at 60°C for 12 h to determine their dry weight. The fecal water content (FWC) was calculated using the following formula:


$$\mathrm{FWC}\left(\%\right)=\frac{\mathrm{wet}\,\mathrm{weight}-\mathrm{dry}\,\mathrm{weight}}{\mathrm{wet}\,\mathrm{weight}}\times 100\%$$

### Gastrointestinal transit rate measurement

Twenty-five minutes after administering activated carbon via gavage, the mice were euthanized and their entire intestinal tract was excised to assess the distance traveled by the charcoal. The Gastrointestinal Transit Rate (GTR) was calculated using the following formula:


$$\mathrm{GTR}\left(\%\right)=\frac{\mathrm{charcoal}\,\mathrm{marker}}{\mathrm{intestinal}\,\mathrm{length}}\times 100\%$$

Then, the thrust is converted to data according to the following formula: $\text{X }=\;\sqrt[{-1}]{\sin p}$. In the formula, *p* represents the Gastrointestinal Transit Rate.

### Small intestine histological examination

Approximately 0.5 cm segments of the small intestine were excised, cleansed with saline, and preserved in 4% paraformaldehyde. These tissue samples were processed for embedding in paraffin and sectioning within 72 h. Following this, they were typically stained using hematoxylin and eosin (H&E). For tissue analysis, an inverted fluorescence microscope (Nikon Eclipse E100, Japan) was employed to evaluate the architectural integrity of intestinal glands, the presence of lymphocyte infiltration, and overall colonic structural changes.

### Biochemical marker quantification

Serum levels of motilin (MTL), substance P (SP), somatostatin (SS), endothelin-1 (ET-1), gastrin (Gas), and vasoactive intestinal peptide (VIP), were assayed using enzyme-linked immunosorbent assay (ELISA) kits provided by Elisa Biotech Co., Ltd. based in Shanghai, China.

### mRNA expression analysis via RT-PCR

Total RNA was extracted from colon tissues using TRIzol reagent (Invitrogen, CA, USA). Subsequently, cDNA was synthesized from 1 μg of RNA as the template, utilizing the RevertAid First Strand cDNA Synthesis Kit (Invitrogen, CA, USA). Real-time PCR was performed on a Bio-Rad system (CA, USA), with GAPDH serving as the housekeeping gene to normalize the data. The primer sequences used in the analysis are summarized in Table 1. The mRNA expression levels were quantified using the 2^–ΔΔCT^ method, which enables the assessment of fold changes in expression relative to the control group.

**TABLE 1:** Primer sequences used in this study.

Gene name	Forward primer sequence	Reverse primer sequence
SCF	5′-CATGGAAGAAAA CGCACCGA-3′	5′-CTTTCCCTTTCTCG GGACCT-3′
c-kit	5′-GACCCGACGCAA CTTCCTTA-3′	5′-TGAGCATCTTCA CGGCAACT-3′
GAPDH	5′-TGTGTCCGTCG TGGATCTGA-3′	5′-TTGCTGTTGAAG TCGCAGGAG-3′

### Measurement of the short-chain fatty acids by UPLC-ESI-MS/MS

Pretreatment: Approximately 50 mg of fecal sample was weighed and mixed with 300 μL of acetonitrile solution (pre-cooled to 4°C) containing internal standards [^2^H_9_]-Pentanoic acid and [^2^H_11_]-Hexanoic Acid. The mixture was ground for 3 min at −20°C, followed by ultrasonic extraction in an ice-water bath for 10 min. The extract was centrifuged at 12,000 rpm for 10 min at 4°C, and the supernatant was collected. The supernatant was diluted 5-fold with acetonitrile solution containing the same internal standards.

Derivatization: An 80 μL aliquot of the diluted supernatant was sequentially mixed with 40 μL of 200 mM 3-NPH solution (prepared in 50% acetonitrile-water, v/v) and 40 μL of 120 mM EDC-6% pyridine solution (prepared in 50% acetonitrile-water, v/v). The reaction was carried out at 40°C for 30 min and subsequently cooled on ice for 1 min. The reaction mixture was filtered through a 0.22 μm microporous membrane to obtain the final derivative solution. The standard solutions were processed in the same manner as the samples.

UPLC-ESI-MS/MS Analysis: Chromatographic Conditions: The injection volume was 1 μL. The mobile phase consisted of solvent A (0.1% formic acid aqueous solution) and solvent B (acetonitrile/methanol, 2:1, v/v), with a flow rate of 0.4 mL/min. The gradient elution program was as follows: 0–2 min, 75% A; 2–11 min, 75% A → 45% A; 12–13 min, 25% A. Mass Spectrometry Conditions: Detection was performed in negative ion mode with a spray voltage of −4,500 V and an ion source temperature of 450°C. The column temperature was maintained at 40°C. Curtain gas pressure was set at 35 psi, while both nebulizing gas (Gas1) and auxiliary gas (Gas2) pressures were set at 50 psi. The collision-induced dissociation (CAD) parameter was set to medium.

### Analysis of the microbial diversity

In the context of this study, a suite of 30 mouse samples was analyzed, yielding a robust dataset with a genomic range spanning 11.19 to 12.43 gigabases. The assemblage of these samples resulted in N50 contig lengths varying between 3,710 and 14,035 base pairs. A comprehensive gene catalog, devoid of redundancy, was constructed from these assemblies, comprising a total of 1,623,375 open reading frames (ORFs). The sample libraries were subjected to sequencing on the state-of-the-art Illumina Novaseq 6000 platform, generating 150-base-pair paired-end reads. Quality control and trimming were meticulously performed using Trimmomatic (version 0.36). Host genome alignment and read filtering were conducted with bowtie2 (version 2.2.9), preceding metagenome assembly via MEGAHIT (version 1.1.2), which led to the reported N50 contig statistics. ORF prediction was executed on the assembled contigs using prodigal (version 2.6.3), and the resulting amino acid sequences were derived. Redundancy was minimized through the application of CDHIT (version 4.5.7), establishing a non-redundant gene set. This set was then aligned to the NR database for taxonomic classification and functionally annotated against various databases using DIAMOND (version 0.9.7). Carbohydrate-active enzymes were identified by comparing the gene sets to the CAZy database with hmmscan (version 3.1), and their activities were approximated by summing gene abundances. Non-metric multidimensional scaling (NMDS) analysis, facilitated by R software (version 3.2.0), was employed to scrutinize the taxonomic and functional abundance profiles across the 30 samples.

### Statistical evaluation methodology

Statistical analysis was performed using SPSS software (version 25.0). For normally distributed and homogeneous variance data, group differences were analyzed using one-way analysis of variance (ANOVA) followed by Tukey’s *post-hoc* test. For other non-normally distributed data, the Kruskal-Wallis test was used to assess differences between groups. Except for differences in species-level taxa within the gut microbiota, which were analyzed using the Kruskal-Wallis test due to their non-normal distribution, all other parameters, including constipation-related phenotypic parameters, gut peptides, cytokine levels, mRNA expression levels, and short-chain fatty acid concentrations, were analyzed using one-way analysis of variance (ANOVA) followed by Tukey’s *post-hoc* test. Graphs were generated using GraphPad Prism (version 9.5). Statistical significance was set at *P* < 0.05.

## Results

### TG11 prevented loperamide-induced constipation in mice

**FIGURE 2:**
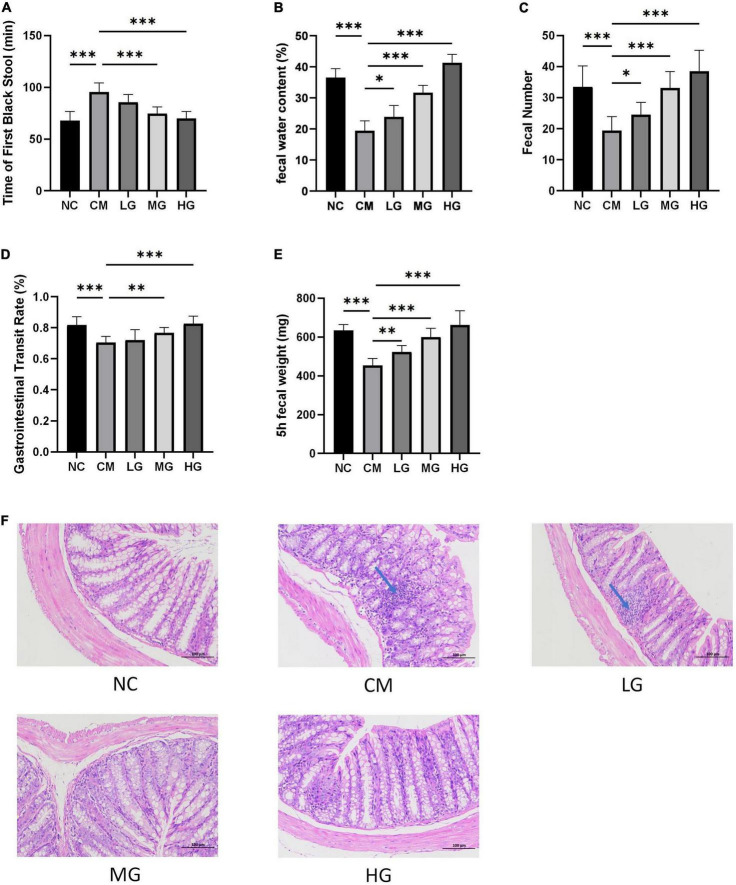
TG11 reduces loperamide-induced constipation in mice. **(A)** Time to First Black Stool: This metric measures the delay in defecation, indicating constipation severity. **(B)** Fecal Water Content: Assesses stool hydration, important for understanding stool consistency. **(C)** Fecal Number: Counts the stools produced over a specific time, indicating bowel movement frequency. **(D)** Gastrointestinal Transit Rate: Measures the efficiency of the digestive tract in moving contents. **(E)** 5-h Fecal Weight: This is similar to **(C)** but limited to a 5-h window, providing insight into short-term effects of TG11. **(F)** Colon Sections Stained with Hematoxylin and Eosin: Visual assessment of intestinal tissue health and response to treatments. Mice were categorized into five groups: a normal control group (NC), a constipation model group (CM), and three groups treated with TG11 at low (LG), medium (MG), and high (HG) doses. Data are presented as mean ± SD. Statistical analysis was performed using one-way ANOVA followed by Tukey’s *post-hoc* test with each treatment group against the model group. The results show significant improvements in TG11-treated groups, with significance levels marked as *P < 0.05, **P < 0.01, ***P < 0.001.

After administering TG11 for 14 days and a single dose of loperamide, the study assessed TG11’s effect on constipation. The findings revealed a significant decrease in the time to the first black stool (F = 22.59, *P* < 0.05), and increase in both fecal water content (F = 89.78, *P* < 0.05) and fecal quantity (F = 5.39, *P* < 0.05) in constipated mice, as depicted in Figures 2A–C. Additionally, the gastrointestinal transit rate (GTR) and 5-h Fecal Weight were markedly lower in the constipation model group (CM) compared to the normal control (NC). TG11 treatment raised GTR (F = 14.35, *P* < 0.05) and 5-h Fecal Weight (F = 34.17, *P* < 0.05) significantly (Figures 2D, E). These results confirm that TG11 effectively alleviates constipation in mice.

The histological assessment using Hematoxylin and Eosin staining revealed the impact of TG11 on the intestinal morphology in mice suffering from constipation. Loperamide treatment at low doses was observed to lead to the disappearance of intestinal glandular structures within the colonic lamina propria, coupled with a significant decrease in lymphocyte infiltration. In contrast, therapy with TG11 was able to maintain the architectural integrity of the intestinal glands in the lamina propria. Furthermore, TG11 exhibited an inhibitory effect on lymphocyte infiltration in the intestinal tract (as illustrated in Figure 2F). However, it should be noted that at low doses, the therapeutic effects of TG11 on preserving colonic structural integrity were less pronounced in mice with constipation.

### Effect of TG11 on the levels of peptide hormones and cytokines in serum of mice

**FIGURE 3:**
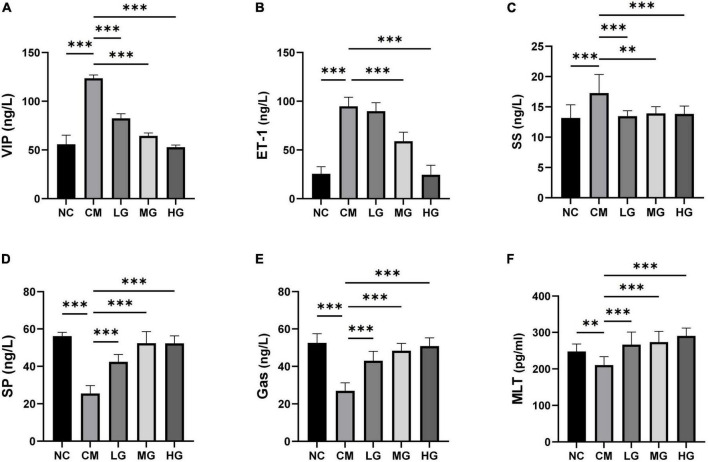
Impact of TG11 on serum levels of peptide hormones and cytokines in mice. **(A)** Vasoactive Intestinal Peptide (VIP): Examines changes in VIP levels, which influence smooth muscle activity and intestinal blood flow. **(B)** Endothelin-1 (ET-1): Focuses on ET-1, a potent vasoconstrictor that affects vascular tone and blood pressure. **(C)** Somatostatin (SS): Assesses levels of SS, which inhibits the secretion of various gastrointestinal hormones. **(D)** Substance P (SP): Tracks SP, involved in pain transmission and inflammation. **(E)** Gastrin (Gas): Gauges the concentration of gastrin, a hormone that stimulates acid secretion in the stomach. **(F)** Mouse Motilin (MTL): Looks at MTL, which plays a role in gastrointestinal motility. The data are expressed as mean ± SD. Statistical analysis was performed using one-way ANOVA followed by Tukey’s *post-hoc* test to compare each treatment group against the model group. Significance levels are indicated with **P < 0.01, ***P < 0.001, highlighting significant changes in hormone and cytokine levels due to TG11 treatment.

To evaluate the therapeutic potential of TG11 in modulating intestinal function through the regulation of critical neurotransmitters and hormones, we conducted quantification of mouse motilin (MTL), substance P (SP), somatostatin (SS), endothelin-1 (ET-1), gastrin (Gas), and vasoactive intestinal peptide (VIP). The constipation group exhibited significantly diminished levels of MTL, SP, and Gas, along with marked elevations in SS, ET-1, and VIP, as compared to the normal control group (Figures 3A–F). Administration of TG11, however, led to a notable restoration of MTL (F = 10.73, *P* < 0.05), SP (F = 35.93, *P* < 0.001), and Gas (F = 42.19, *P* < 0.001) to near physiological levels, concomitant with a significant reduction in SS (F = 6.23, *P* < 0.05), ET-1 (F = 114.93, *P* < 0.001) and VIP (F = 170.81, *P* < 0.001) concentrations. These data imply that TG11 may represent a promising intervention for the management of slow transit constipation, by re-equilibrating the intestinal neurotransmitter and hormone profile.

### Effects of TG11 on mRNA levels of constipation-related intestinal factors

**FIGURE 4:**
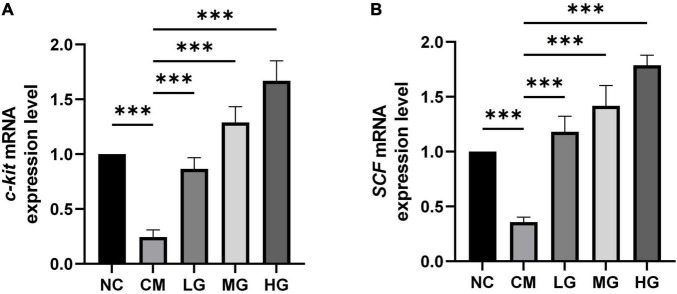
Effects of TG11 on mRNA levels of constipation-related intestinal factors. **(A)** mRNA Expression Levels of *SCF*: This panel measures the mRNA levels of *Stem Cell Factor* (*SCF*), which plays a role in cellular signaling and motility within the intestinal tract. **(B)** mRNA Expression Levels of *c-kit*: Explores the mRNA levels of *c-kit*, a receptor essential for the function and survival of certain cell types in the gastrointestinal system. The results are expressed as mean ± SD. Statistical analysis was conducted using one-way ANOVA followed by Tukey’s *post-hoc* test, comparing the control and intervention groups with the model group. Significance levels are marked as ***P < 0.001, indicating significant changes in the mRNA levels of *SCF* and *c-kit* due to TG11 treatment.

Studies have indicated that the mRNA expression levels of *SCF* and *c-kit* are associated with intestinal function. Constipation may be related to weakened intestinal motility and abnormal intestinal mucosal function. A decrease in the mRNA expression of *SCF* or *c-kit* could lead to insufficient activation of the c-kit receptor, thereby affecting intestinal motility and secretory functions, potentially exacerbating constipation symptoms. In mice with constipation, the mRNA expression levels of *SCF* and *c-kit* in the colon were significantly reduced. Compared to the control group (CM), treatment with various doses of TG11 increased the expression of *SCF* (F = 119.44, *P* < 0.001) and *c-kit* (F = 119.44, *P* < 0.001) by two to threefold, thereby alleviating the symptoms of constipation (Figures 4A, B).

### Effect of TG11 on the short-chain fatty acids content in feces of mice

**FIGURE 5:**
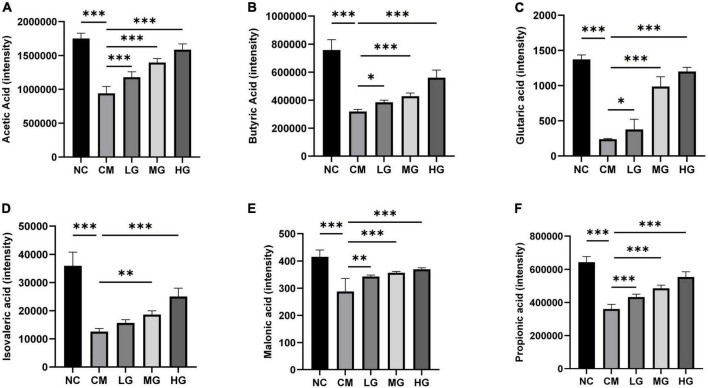
Effect of TG11 on the short-chain fatty acids content in feces of mice. **(A)** Acetic Acid **(B)** Butyric Acid **(C)** Glutaric acid **(D)** Isovaleric acid **(E)** Malonic acid **(F)** Propionic acid. The results are expressed as mean ± SD. Statistical analysis was conducted using one-way ANOVA followed by Tukey’s *post-hoc* test, comparing the control and intervention groups with the model group. Significance levels are marked as *P < 0.05, **P < 0.01, ***P < 0.001, indicating significant changes in the short-chain fatty acids (SCFAs) in fecal specimens due to TG11 treatment.

Utilizing Ultra-Performance Liquid Chromatography-Electrospray Ionization Tandem Mass Spectrometry (UPLC-ESI-MS/MS), we investigated the impact of TG11 on the Short-Chain Fatty Acids (SCFAs) profile in fecal samples. The study comprised five cohorts: Control, Constipation Model (CM), and Low-, Medium-, and High-dose TG11 treatment groups. Our findings revealed a significant reduction in various SCFAs, including acetic (F = 77.58, *P* < 0.001), butyric (F = 86.49, *P* < 0.001), glutaric (F = 94.85, *P* < 0.001), isovaleric (F = 59.31, *P* < 0.001), malonic (F = 67.27, *P* < 0.001), and propionic acids (F = 14.25, *P* < 0.001), in the CM group compared to the Control. This observation suggests a substantial alteration in the metabolic functionality of the gut microbiota in the constipation model, resulting in diminished SCFA production. Intriguingly, we observed a dose-dependent restoration of SCFA levels with TG11 treatment. As the TG11 dosage increased, SCFA concentrations progressively recovered, converging toward those observed in the Control group. This trend implies a potential therapeutic effect of TG11 in normalizing gut microbiota metabolism and SCFA production in constipation (Figures 5A–F). In addition to the six SCFAs mentioned above, we also detected six other SCFAs, including pentanoic acid, hexanoic acid, 2,3-dihydroxy-3-methylbutanoic acid, lactic acid, malonic acid, and glutaric acid. However, these SCFAs did not show statistically significant differences between groups (*P* > 0.05).

### Effect of TG11 on the structure of gut microbiota in mice

**FIGURE 6:**
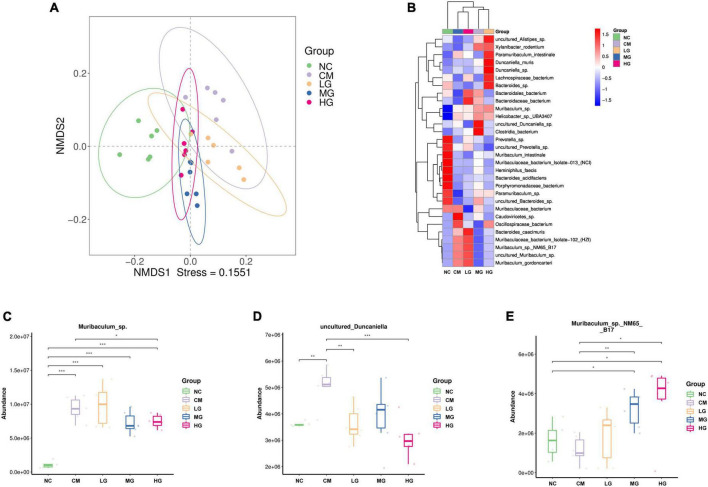
Impact of TG11 on the composition of gut microbiota in mice. **(A)** NMDS analysis at the species level **(B)** Heatmap of abundance of species-level bacteria in mice. **(C)** the abundance of *Muribaculum*_sp **(D)** the abundance of *Duncaniella* **(E)** the abundance of *Muribaculum*_sp_NM65_B17. Kruskal-Wallis test was conducted to compare the control and strain groups with the model group; *P < 0.05, **P < 0.01, ***P < 0.001.

The NMDS analysis at the species level indicated that the NC group was distinct from the CM group, whereas the LG group showed a close relationship with the CM group. The MG and HG groups were also similar to the CM group, indicating that medium and high doses of TG11 can maintain a balanced state of the intestinal microbiota in mice (Figure 6A). We observed that higher doses of TG11 were linked to significant alterations in the composition of the gut microbiota, particularly at high doses (Figure 6B). At the species level, loperamide increased the abundance of *Muribaculum*_sp. (*P* < 0.05) and *uncultured_Duncaniella* (*P* < 0.05), while reducing the abundance of *Muribaculum* sp._NM65 B17, an effect that was reversed by TG11 treatment (Figures 6D–E).

### TG11’s effect on mouse biological pathways

**FIGURE 7:**
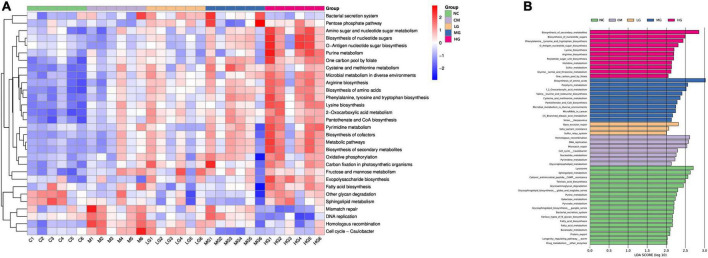
Differential effects of TG11 on mouse biochemical pathways. **(A)** Heatmap of the top 30 KEGG level 3 pathways with significant differences as determined by Kruskal-Wallis analysis, without clustering **(B)** Results from the LEfSe analysis of KEGG level 3 pathways.

In our KEGG pathway analysis, we detected substantial disparities in a multitude of biochemical pathways between the TG11 intervention groups at varying doses, the disease model group, and the control. These pathways include, but are not limited to, fatty acid metabolism, drug metabolism, DNA repair, amino acid biosynthesis, and sulfur metabolism (Figure 7A). Notably, the high-dose TG11 intervention group (HG) exhibited pronounced alterations in pathways related to amino acid synthesis, neurogrowth factor signaling, and angiogenesis, implying a regulatory function of TG11 in these physiological processes. The moderate-dose (MG) and low-dose (LG) groups also revealed dose-dependent changes in select KEGG pathways, further highlighting the gradient of TG11’s impact (Figure 7B).

## Discussion

Constipation, a gastrointestinal disorder that affects millions of people worldwide, severely reduces the quality of life of sufferers (Bharucha and Lacy, 2020). The condition is characterized by reduced bowel movements, hard stool consistency, and difficulty in defecation (Heidelbaugh et al., 2021). Currently, laxatives or softeners are commonly used for treatment, but their efficacy is limited and they come with side effects, leading to increased interest in the use of probiotics as a side-effect-free therapy (Lacy et al., 2021). Our research indicates that the probiotic strain TG11 effectively alleviates constipation induced by loperamide. Mechanistically, TG11 acts on multiple aspects including gastrointestinal peristalsis, intestinal morphology, and the composition of the gut microbiota.

The SCF/c-kit signaling pathway is involved in the regulation of intestinal peristalsis and mucosal function. The binding of the SCF (stem cell factor) ligand to the c-kit receptor activates downstream signaling pathways, modulating the release of gastrointestinal peptides and neurotransmitters (Zhang et al., 2024). In constipation, the mRNA expression of this pathway is downregulated, which suppresses intestinal peristalsis and fecal transit (Lennartsson and Rönnstrand, 2012). TG11 significantly increased the mRNA expression levels of *SCF* and *c-kit* in colonic tissue, The SCF/c-kit signaling pathway is not unique to smooth muscle cells. This pathway is also expressed in mast cells and interstitial cells of Cajal (Chai et al., 2017; Rönnstrand, 2004). The changes in *SCF* and *c-kit* expression levels observed in this study, which used colonic tissue for measurement, may be a combined effect of various cell types. This is one of the limitations of our study. Future research could consider using cell-specific methods to clarify the responses of the SCF/c-kit signaling pathway to TG11 treatment in different cell types. Mice gastrin (MTL) can promote gastrointestinal peristalsis and gastric emptying (Duan et al., 2021), while substance P (SP) is involved in pain signal transmission and intestinal peristalsis (Ray et al., 2009), somatostatin (SS) inhibits gastrointestinal secretion and peristalsis (De Martino et al., 2010), endothelin-1 (ET-1) constricts blood vessels, regulates blood pressure, and participates in inflammatory responses (Houde et al., 2016), gastrin (Gas) promotes gastric acid secretion and gastrointestinal peristalsis (Norton et al., 2018), and vasoactive intestinal peptide (VIP) dilates blood vessels and regulates intestinal peristalsis and secretion (Iwasaki et al., 2019). After constipation, the altered levels of these peptides and cytokines lead to impaired intestinal function. Our study shows that TG11 treatment restores these indicators to near-physiological levels, and this restoration becomes more significant with increasing dosage. Amino acid metabolism is associated with intestinal peristalsis, gut microbiota balance, and intestinal osmotic pressure (Li et al., 2024; Wu et al., 2021). Branched-chain amino acids (BCAAs), after metabolism in the intestine, produce short-chain fatty acids that stimulate intestinal peristalsis and promote defecation (Choi et al., 2024). Tyramine produced in the metabolic pathways of phenylalanine and tyrosine may affect the function of the intestinal nervous system, thereby influencing intestinal peristalsis (Fernstrom and Fernstrom, 2007). KEGG analysis results suggest that amino acid metabolism may be an important pathway for the action of TG11, with the highest dose being the most significant.

We found that a dose of 4 mg/kg of loperamide was sufficient to induce constipation in mice, thus we adopted this dose, which is different from the commonly used dose of 10 mg/kg in other studies (Jang et al., 2024; Zhang et al., 2023b; Zhang et al., 2023a). We utilized high-resolution metagenomic sequencing techniques to analyze the gut microbiota, a method that can directly identify bacteria to the species level (Wang et al., 2024). *Muribaculum* can regulate the barrier function of the intestinal mucosa and immune response, while *Duncaniella* protects the intestinal barrier and is involved in the production of short-chain fatty acids (SCFAs) (Chang et al., 2021; Choi et al., 2021). TG11 significantly increased the levels of short-chain fatty acids, including Acetic Acid, Butyric Acid, Glutaric acid, Isovaleric acid, Malonic acid, and Propionic acid. Acetic acid, propionic acid, butyric acid, and isovaleric acid are mainly produced by gut microbiota (Hays et al., 2024; Muller et al., 2021), while glutaric acid and malonic acid are primarily derived from the tricarboxylic acid (TCA) cycle (Arnold and Finley, 2023). This suggests that the TG11 strain not only promotes the production of short-chain fatty acids by regulating gut microbiota but may also increase short-chain fatty acid content by influencing the host’s energy metabolism pathways. In our study, the abundance of *Muribaculum*_sp and *Duncaniella* was significantly elevated in the CM group, and TG11 treatment restored their abundance to the level of the NC group, indicating that these two bacteria may help the body against constipation. The abundance of *Muribaculum*_sp_NM65_B17 was reduced in the CM group but significantly increased in all dose treatment groups with TG11, suggesting that TG11 may contribute to the growth of this bacterium.

Recent studies have increasingly focused on *Bifidobacterium* in the field of constipation research. Similar to our study, Fu et al. (2024) used a loperamide-induced mouse model to investigate the therapeutic effects of NKU FB3-14 on constipation. Both studies demonstrated the ability to modulate intestinal peptides including MLT, SS, and VIP. NKU FB3-14 did not exhibit a regulatory effect on SP levels. Both NKU FB3-14 and TG11 significantly increased the butyrate content in the feces of constipated mice. Moreover, TG11 also elevated the levels of acetate, propionate, isovaleric acid, glutaric acid, and malonic acid in the feces. In the constipation model established by Fu et al. (2024), the abundance of *Ruminococcaceae* and *Olsenella* decreased, whereas in our model, the abundance of *Muribaculum* sp. and *Duncaniella* increased. This discrepancy could be attributed to several factors. The animal strains used were different: we used ICR mice, while Fu et al. (2024) used BALB/c mice. Different mouse strains may respond differently to the same treatment. The influence on SP levels suggests that the effect of NKU FB3-14 on intestinal motility may be more pronounced in functional constipation rather than organic lesions (Ray et al., 2009). Compared to NKU FB3-14, TG11 exerted a broader influence on SCFAs, indicating that TG11 might promote the growth of gut microbiota that produce SCFAs. In the study by Wang et al. (2022), *Bifidobacterium longum* effectively alleviated constipation in mice induced by loperamide. Similar to TG11, *B. longum* significantly improved all constipation-related indicators but did not ameliorate MTL, SP, SS, and VIP levels. Moreover, *B. longum* only increased the acetate level in feces, differing from the broad effects of TG11 on SCFAs. The differences might stem from the mechanism by which *B. longum* alleviates constipation, which does not rely on gastrointestinal hormones. Additionally, Wang et al. (2022) used BALB/c mice, differing from the ICR strain used in our study. Comparing these two bifidobacteria, we observed that although certain bifidobacteria have the ability to alleviate constipation, their mechanisms of action and effective doses vary. This highlights the need to select suitable probiotics for intervention based on the phenotype of constipation.

A 2006 clinical study confirmed W11’s efficacy in alleviating constipation (Amenta and Cascio, 2006). Subsequently, Di Pierro et al. (2024) through bioinformatics analysis and cultivation experiments, revealed that W11 metabolizes arabinomannan to produce SCFAs, thereby improving intestinal motility–a process closely linked to *abfA* and *abfB* gene expression. In a randomized, triple-blind, placebo-controlled clinical trial, Cheng et al. (2024) found that while HN019 did not significantly increase complete spontaneous bowel movement (CSBM) frequency, it significantly improved abdominal pain and bloating. This suggests that although certain probiotics show promising results in animal studies, further clinical validation is essential.

These findings provide a new perspective on the treatment of constipation, and probiotic therapy holds the promise of improving the quality of life for constipation sufferers. Subsequent studies should further develop the application of probiotics in the treatment of constipation, providing a more solid scientific basis for clinical practice.

## Conclusion

This study investigates the ameliorative effects of the probiotic *Bifidobacterium animalis* subsp. *lactis* TG11 on loperamide-induced constipation in mice, alongside the underlying mechanisms involved. Our findings reveal that TG11 significantly alleviates constipation symptoms, evidenced by a reduction in defecation time, an increase in fecal water content and quantity, and an enhancement in intestinal transit rate. Mechanistic investigations suggest that TG11 exerts its beneficial effects through multiple pathways: (1) modulating the levels of gut-related peptide hormones and cytokines; (2) upregulating the mRNA expression of *SCF* and *c-kit* in colonic tissues; (3) restoring gut microbiota balance; (4) regulating amino acid metabolism across various biological pathways. These insights provide a robust scientific foundation for the development of novel probiotic formulations aimed at treating constipation.

## Data Availability

The original contributions presented in this study are publicly available. This data can be found here: https://www.ncbi.nlm.nih.gov/sra/PRJNA1207417. The object IDs and corresponding URLs can be found in Supplementary Data Sheet 1.
