# Supplementary material for: Bifidobacterium animalis subsp. lactis TG11 ameliorates loperamide-induced constipation in mice by modulating gut microbiota
Source: Front Microbiol. 2025 Jan 29;16:1525887. doi: 10.3389/fmicb.2025.1525887 (PMC11813947; doi:10.3389/fmicb.2025.1525887)
Supplement: Supplementary file 1 [file Data_Sheet_1.docx]

The datasets generated for this study can be found in the NCBI Sequence Read Archive (SRA) under BioProject accession number PRJNA1207417, accessible at https://www.ncbi.nlm.nih.gov/sra/PRJNA1207417

Object IDs and corresponding URLs:

RUN:41655795: https://www.ncbi.nlm.nih.gov/sra/RUN:41655795

RUN:41655794: https://www.ncbi.nlm.nih.gov/sra/RUN:41655794

RUN:41655793: https://www.ncbi.nlm.nih.gov/sra/RUN:41655793

RUN:41655792: https://www.ncbi.nlm.nih.gov/sra/RUN:41655792

RUN:41655791: https://www.ncbi.nlm.nih.gov/sra/RUN:41655791

RUN:41655790: https://www.ncbi.nlm.nih.gov/sra/RUN:41655790

RUN:41655789: https://www.ncbi.nlm.nih.gov/sra/RUN:41655789

RUN:41655788: https://www.ncbi.nlm.nih.gov/sra/RUN:41655788

RUN:41655787: https://www.ncbi.nlm.nih.gov/sra/RUN:41655787

RUN:41655786: https://www.ncbi.nlm.nih.gov/sra/RUN:41655786

RUN:41655785: https://www.ncbi.nlm.nih.gov/sra/RUN:41655785

RUN:41655784: https://www.ncbi.nlm.nih.gov/sra/RUN:41655784

RUN:41655783: https://www.ncbi.nlm.nih.gov/sra/RUN:41655783

RUN:41655782: https://www.ncbi.nlm.nih.gov/sra/RUN:41655782

RUN:41655781: https://www.ncbi.nlm.nih.gov/sra/RUN:41655781

RUN:41655780: https://www.ncbi.nlm.nih.gov/sra/RUN:41655780

RUN:41655779: https://www.ncbi.nlm.nih.gov/sra/RUN:41655779

RUN:41655778: https://www.ncbi.nlm.nih.gov/sra/RUN:41655778

RUN:41655777: https://www.ncbi.nlm.nih.gov/sra/RUN:41655777

RUN:41655776: https://www.ncbi.nlm.nih.gov/sra/RUN:41655776

RUN:41655775: https://www.ncbi.nlm.nih.gov/sra/RUN:41655775

RUN:41655774: https://www.ncbi.nlm.nih.gov/sra/RUN:41655774

RUN:41655773: https://www.ncbi.nlm.nih.gov/sra/RUN:41655773

RUN:41655772: https://www.ncbi.nlm.nih.gov/sra/RUN:41655772

RUN:41655771: https://www.ncbi.nlm.nih.gov/sra/RUN:41655771

RUN:41655770: https://www.ncbi.nlm.nih.gov/sra/RUN:41655770

RUN:41655769: https://www.ncbi.nlm.nih.gov/sra/RUN:41655769

RUN:41655768: https://www.ncbi.nlm.nih.gov/sra/RUN:41655768

RUN:41655767: https://www.ncbi.nlm.nih.gov/sra/RUN:41655767

RUN:41655766: https://www.ncbi.nlm.nih.gov/sra/RUN:41655766
